# Supplementary material for: Impairments in the early consolidation of spatial memories via group II mGluR agonism in the mammillary bodies
Source: Sci Rep. 2024 Mar 12;14:5977. doi: 10.1038/s41598-024-56015-3 (PMC10933409; doi:10.1038/s41598-024-56015-3)
Supplement: Supplementary file 1 — Supplementary Information. [file 41598_2024_56015_MOESM1_ESM.docx]

**Supplementary Materials**

**Supplementary Figures**


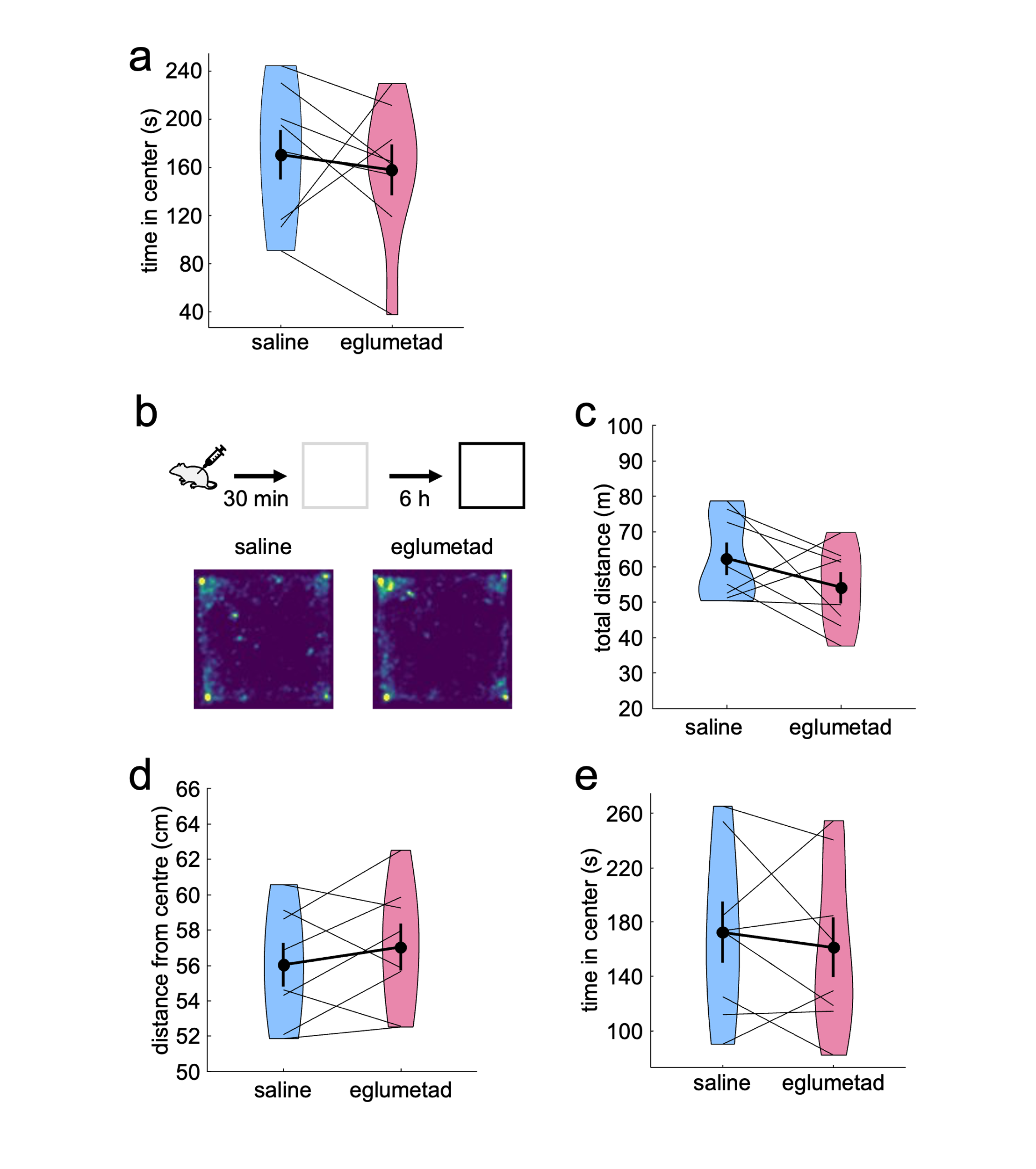


**Fig. S1.** **The effect of eglumetad injections on time in center of the open field after a 30 min delay and on total distance, mean distance from center and time in center after a 6 h delay in the rat**. **b** – Schematic representation of the task and heatmaps showing mean arena occupancy upon a second exposure to the arena 6.5 h from injection (n = 8, within-subject design). **c-e** – Violin plots of the total distance travelled, mean distance from the center of the maze and time in the center of the maze, respectively.


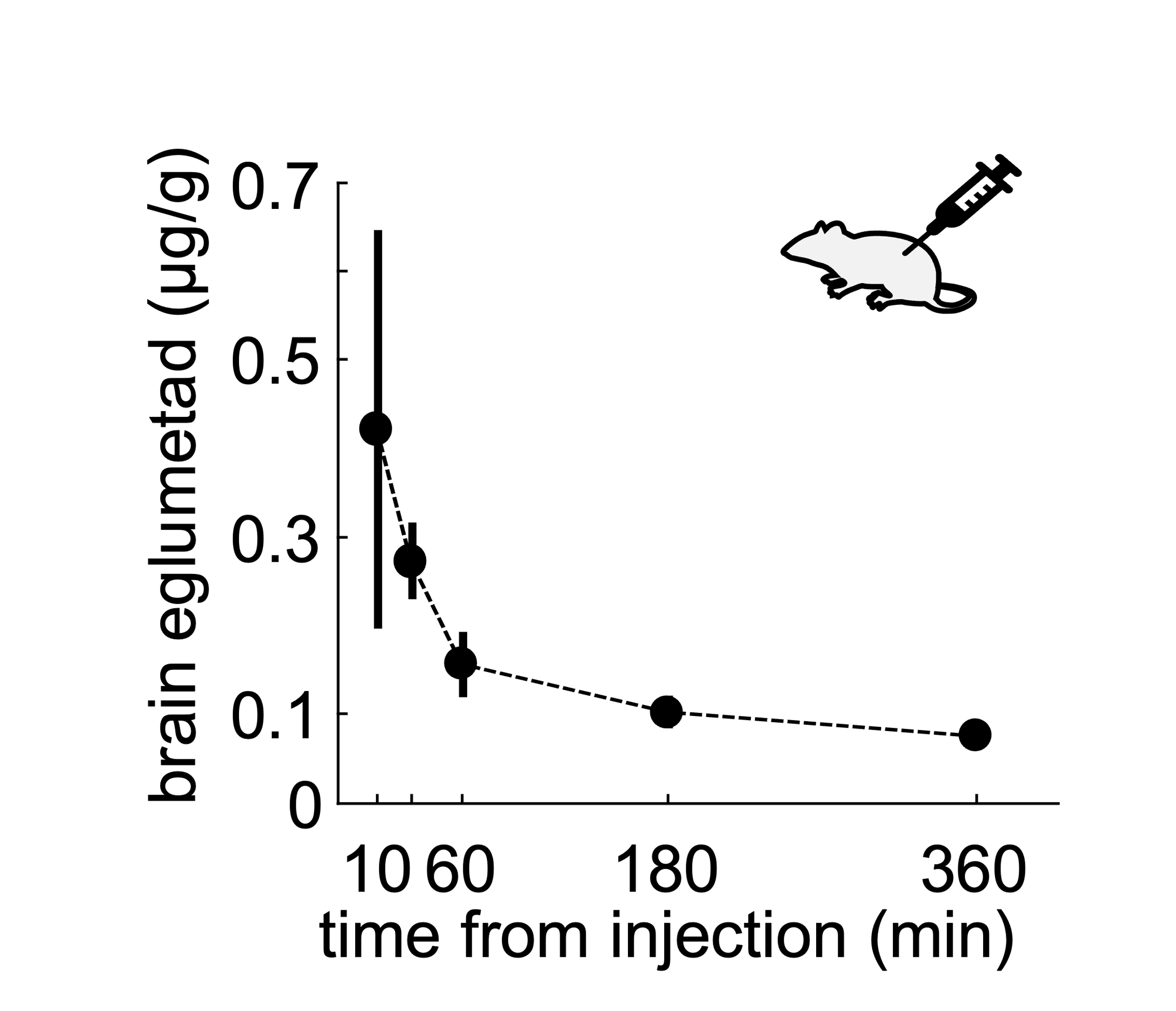


**Fig. S2.** Concentration of eglumetad in rat brain at increasing intervals from intraperitoneal injection (10 min, 30 min, 1 h, 3 h, 6 h). The dots represent means and the vertical bars, the standard error of the mean (n = 20 with one rat serving as blank control).


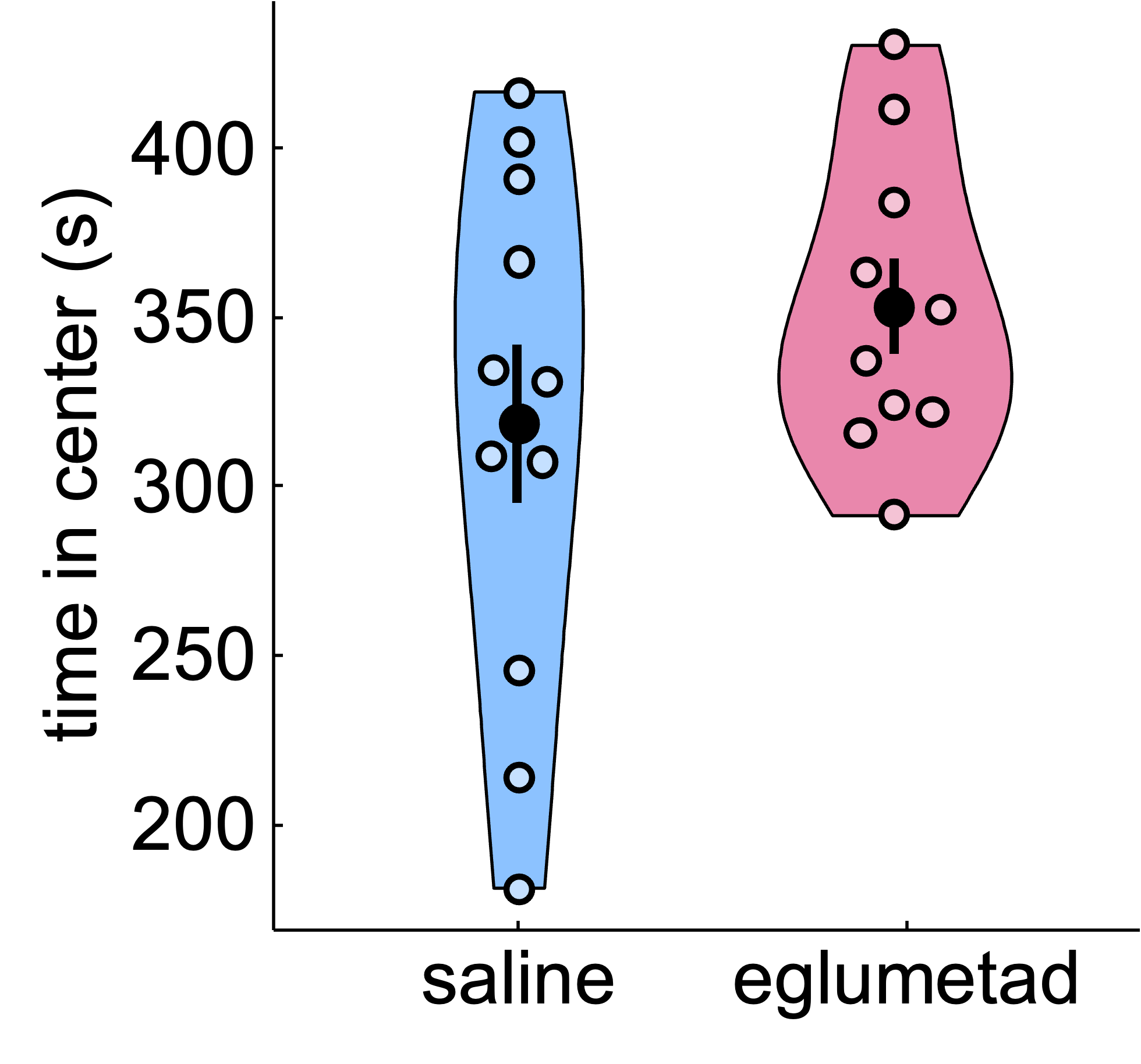


**Fig. S3. The effect of eglumetad injections on time in center of the maze after a 30 min delay in mice.**


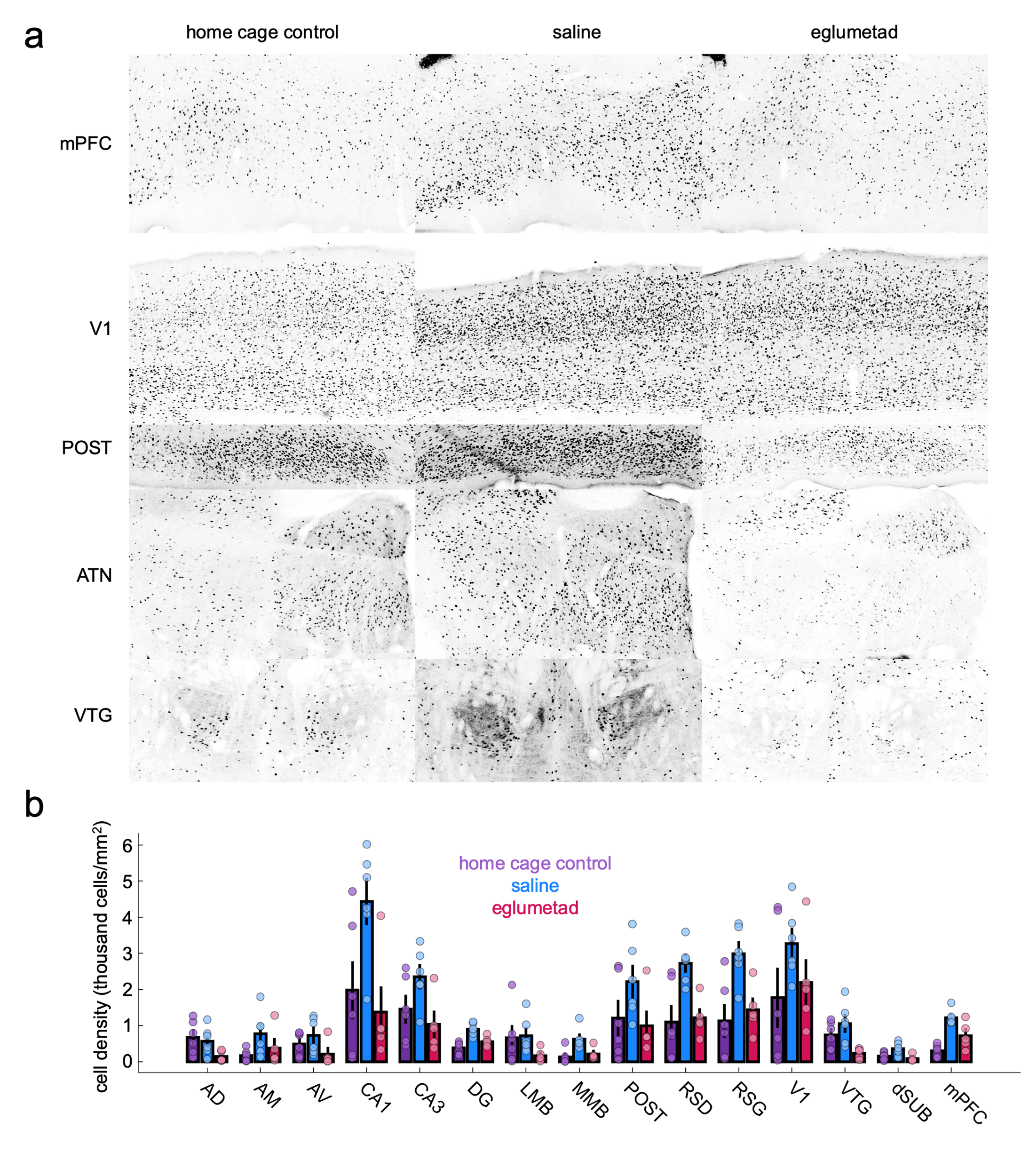


**Fig. S4**. **Raw c-fos expression levels in home. a** – Representative images of regional c-fos expression levels in home cage controls, saline-injected and eglumetad-injected mice. **b** – Quantification of regional cell densities in home cage controls, saline-injected and eglumetad-injected mice. Abbreviations as in **Fig. 3**.


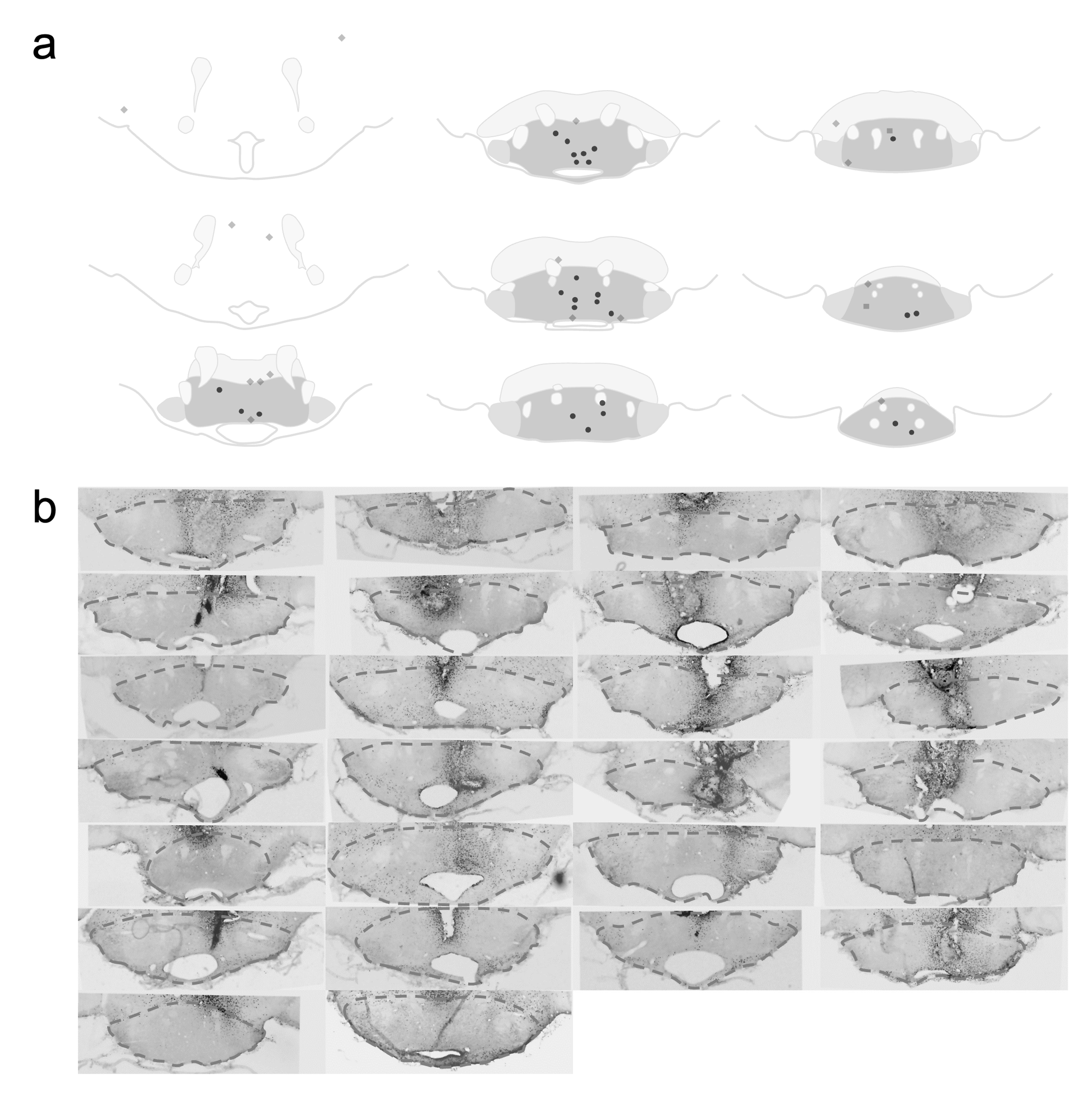


**Fig. S5. Histological validation of local drug infusion.** **a** – Estimated positions of cannula tips across 9 coronal levels. **b** – Images showing cannula tip location. The dark hyperintensities are caused by heightened DAB staining in scarred tissue around implants.


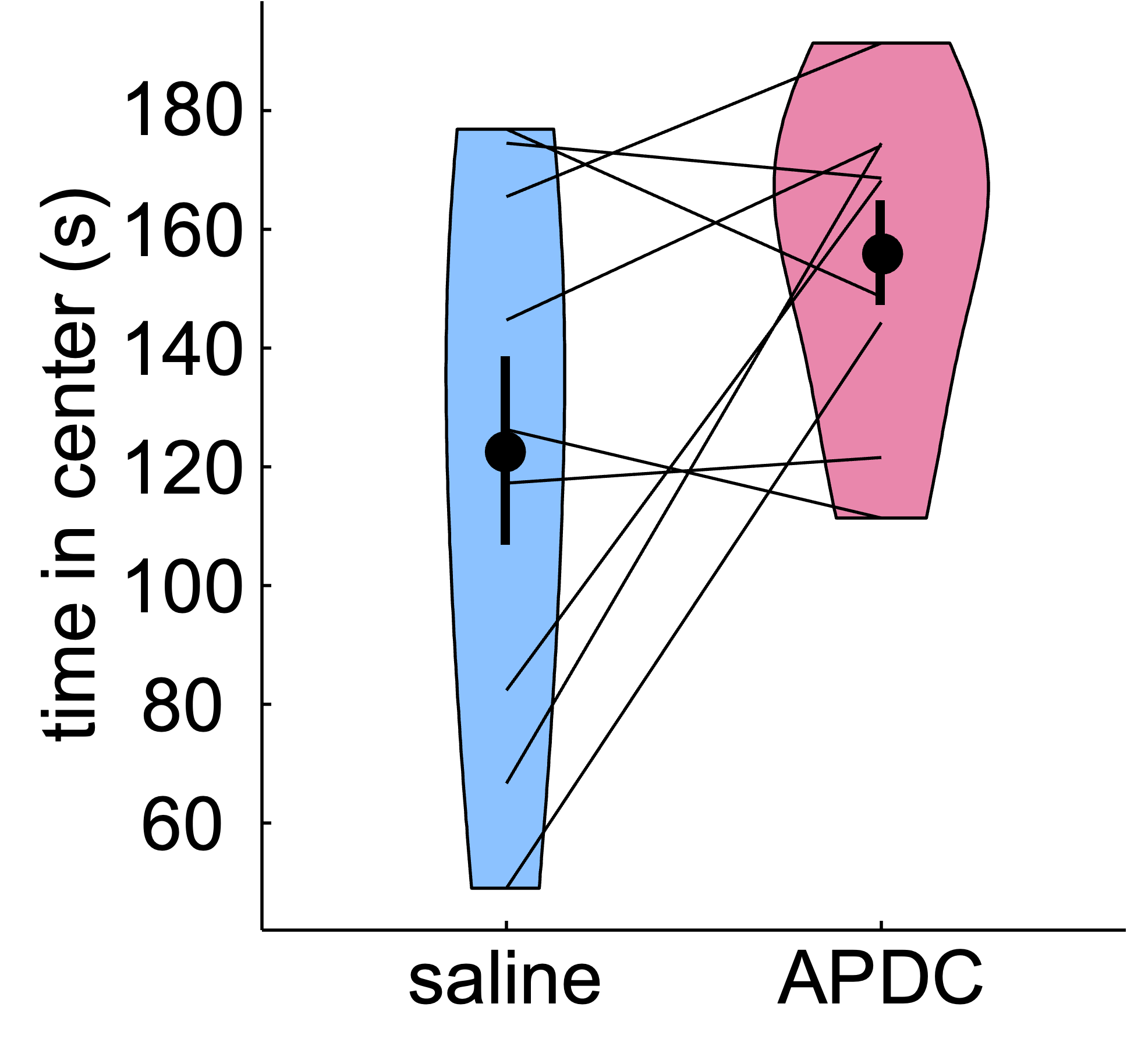


**Fig. S6. The effect of APDC infusion on time in center of the maze after a 15 min delay in rats.**


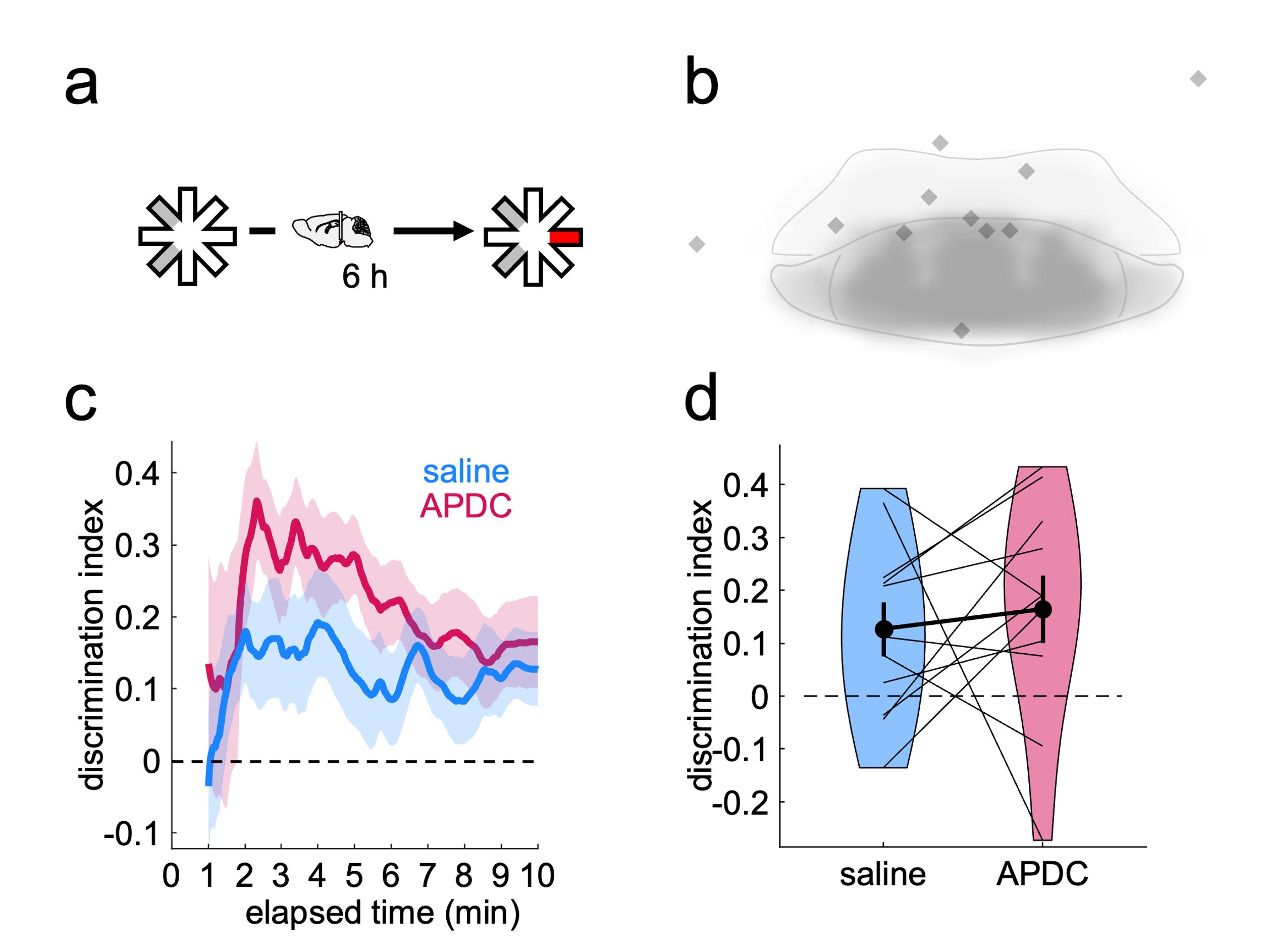


**Fig. S7. The absence of drug effect in miscannulated animals. a** – Schematic representation of the novel place preference task. **b** – Estimated positions of misplaced cannulae. Note, due to fiber encapsulation of the mammillary bodies, infusions made at the dorsal boundaries do not spread into the mammillary bodies. **c** – Timeline of cumulative discrimination for miscannulated animals (n = 11; excludes two cases removed from analyses due to poor sample exploration activity, see Methods). **d** – Violin plots of cumulative discrimination scores at 10 min of test exploration.

**Supplementary Table 1**

**Comparison of permutation-based and parametric test results for behavioral experiments.**


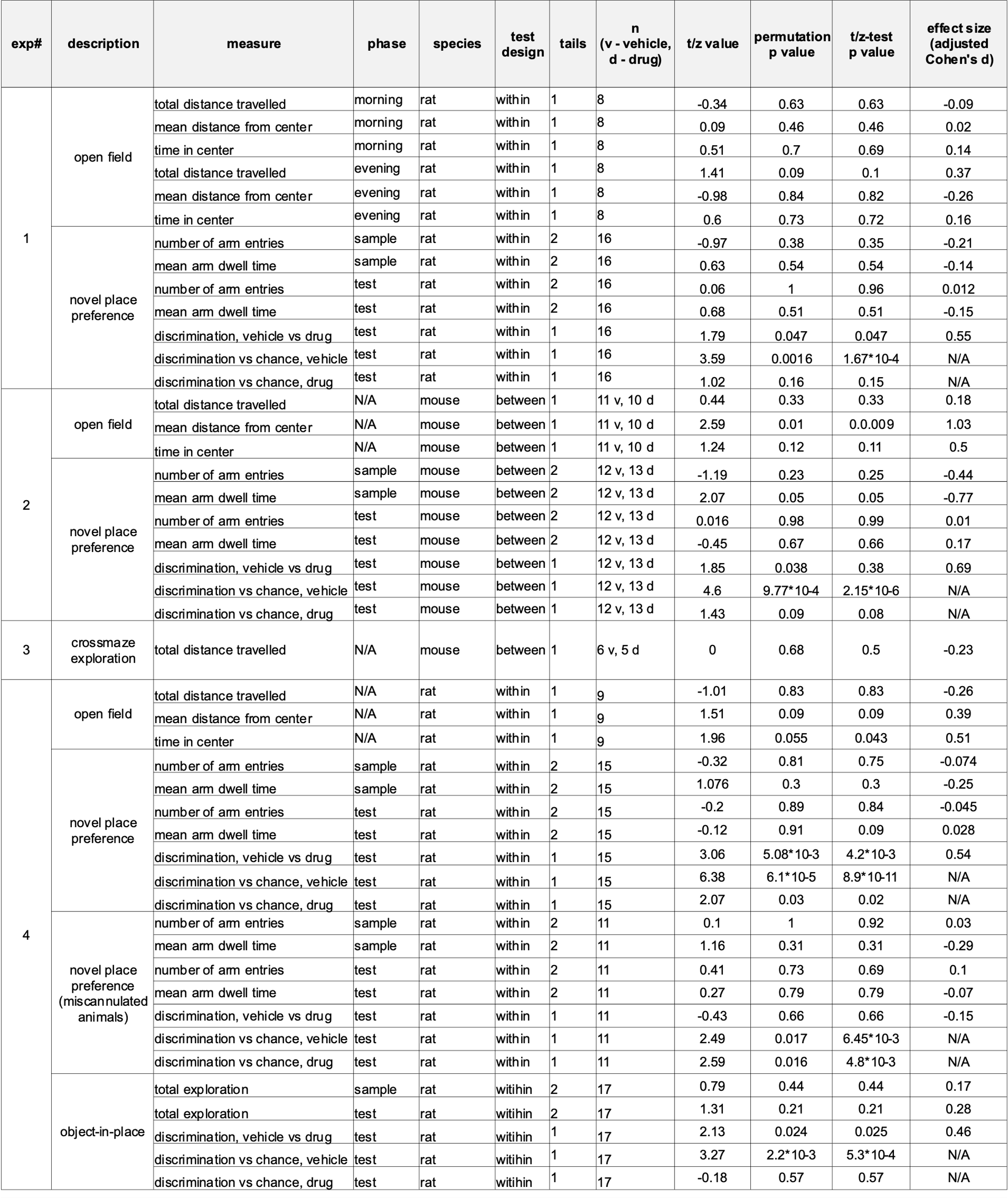


**Supplementary Methods for Brain Occupancy Analysis**

Liquid chromatography–mass spectrometry analysis: Analyses were carried out using the Acquity UPLC system (Waters Corporation, USA). 20 µL of each sample (tested in duplicate) were subjected to the following chromatography: Solvent A - Milli-Q water + 0.2% Acetonitrile + 0.1 % formic acid (F.A.); Solvent B – Acetonitrile + 0.1 % F.A.; Flow rate – 0.5 mL/min; Column (Waters HSS T3, 2.1 x 50 mm, 1.8 µm particle); Guard Column - ACQUITY UPLC HSS T3 VanGuard Pre-column, 100Å, 1.8 µm, 2.1 mm X 5 mm, (Waters, cat # 186003976).

Gradient (5 min):

| Time (min) | Solvent A % | Solvent B % | Curve |
| --- | --- | --- | --- |
| 0 | 100 | 0 | 1 |
| 0. 5 | 100 | 0 | 11 |
| 2.5 | 0 | 100 | 6 |
| 5 | 100 | 0 | 1 |

Mass spectrometry analysis

|  | Quattro Premier | Xevo TQs-Micro |
| --- | --- | --- |
| Capillary voltage (kV) | 3 | 2.5 |
| Cone voltage | Defined in the MS method | |
| Source temp | 120 °C | - |
| Desolvation temp | 300 °C | 450 °C |
| Desolvation gas | 800 L/hr | 800 L/hr |
| Cone gas | 30 L/hr | 30 L/hr |
| MS 1 resolution (LM) | 15 | 15 |
| MS 1 resolution (HM) | 15 | 15 |
| MS 1 ion energy | 0.2 | 0.1 |
| Collision cell entrance | 0.2 | - |
| Collision cell exit | 0.4 | - |
| Collision energy | Defined in the MS method | |
| MS 2 resolution (LM) | 13 | 14 |
| MS 2 resolution (HM) | 13 | 14 |
| MS 2 ion energy | 0.1 | 0.1 |
